# Lifestyle Variations during and after the COVID-19 Pandemic: A Cross-Sectional Study of Diet, Physical Activities, and Weight Gain among the Jordanian Adult Population

**DOI:** 10.3390/ijerph19031346

**Published:** 2022-01-25

**Authors:** Hanan Hammouri, Fidaa Almomani, Ruwa Abdel Muhsen, Aysha Abughazzi, Rawand Daghmash, Alaa Abudayah, Inas Hasan, Eva Alzein

**Affiliations:** 1 Department of Mathematics and Statistics, Jordan University of Science and Technology, Irbid 22110, Jordan; rmabedalmohssen17@sci.just.edu.jo; 2 Department of Rehabilitation Sciences, Jordan University of Science and Technology, Irbid 22110, Jordan; falmomani@just.edu.jo; 3 Department of English Language and Linguistics, Jordan University of Science and Technology, Irbid 22110, Jordan; aabughazzi@just.edu.jo; 4 Department of Pharmaceutical Technology, Jordan University of Science and Technology, Irbid 22110, Jordan; rmdaghmash19@ph.just.edu.jo (R.D.); aaabudayah19@ph.just.edu.jo (A.A.); iihasan19@ph.just.edu.jo (I.H.); 5 Department of Public Health, Jordan University of Science and Technology, Irbid 22110, Jordan; eva.alzain@jfda.jo

**Keywords:** COVID-19 pandemic, dietary habits, BMI, nutrition, family, household, healthy food, dietary supplements intake, lifestyle, weight gain

## Abstract

The way that COVID-19 has been handled since its inception in 2019 has had a significant impact on lifestyle-related behaviors, such as physical activities, diet, and sleep patterns. This study measures lifestyle-related behavior during the COVID-19 pandemic lockdown using a 22-item questionnaire. The responses were collected from March 2021 to September 2021. A total of four hundred and sixty-seven Jordanian participants were engaged in assessing the changes caused by the pandemic and their effect on BMI. The validity and reliability of the questionnaire were tested for 71 participants. Cronbach’s alpha values for the questionnaire exceeded 0.7, demonstrating good reliability and internal consistency. The effect of each question regarding physical activity and dietary habits over the BMI difference was studied using ANOVA. The study shows that more than half of the participants reported snacking more between meals and increased their sitting and screen time, while 74% felt more stressed and anxious. BMI difference among the individuals throughout the lockdown was significantly associated with these variables. In contrast, 62% of the participants showed more awareness about their health by increasing the intake of immunity-boosting foods, and 56% of the participants showed an increase in the consumption of nutrition supplements. Females and married individuals tended to be healthier. Therefore, their BMI showed stability compared to others based on their gender and marital status. Exercise, sleep, and avoiding ‘junk’ food, which contributes to weight gain and COVID-19 vulnerability, are strongly recommended.

## 1. Introduction

Throughout history, humanity has experienced several pandemics and diseases that affected lives and caused massive infections and deaths, such as the Cyprian Plague in 250 AD, Leprosy in the 11th century, and the Black Death in 1350. Respiration-related pandemics include the Russian and Spanish Flu in 1889 and 1918, respectively, and severe acute respiratory syndrome (SARS) in 2003, ending with COVID-19 in 2019 until the present [1,2]. As a result of these pandemics, human health, lifestyles, and social lives were profoundly affected. Moreover, economies both local and global were affected [1].

Several researchers have studied the effects of these pandemics. For instance, Horgan [3] mentioned that the Cyprian Plague caused political turbulence as the outbreak claimed the lives of two emperors: Claudius II Gothicus in 270 CE and Hostilian in 251 CE. Moreover, turbulence in the economic situation appeared as the farmers moved to cities instead of farms because of the spread of the pandemics in the rural areas, which minimized agricultural production, leading to instability in the economic situation.

Black Death also had a massive economic effect in 1350. North Africa, mainland Italy, Spain, France, Austria, Hungary, Switzerland, Germany, and several countries went into extreme inflation because it was hazardous to procure goods through external traders, and it was challenging to produce goods due to the massive number of deaths among workers and farmers. Therefore, the prices of both goods produced locally and those imported from afar skyrocketed [4]. In 2003, SARS emerged, which infected 8096 people in 29 countries, and 774 died. Moreover, during the SARS outbreak, China’s growth decreased in the first quarter of 2003 from 11.1% to 9.1% [5].

Worldwide, humanity recently suffered from a SARS-modified virus named Coronavirus disease (COVID-19). The first case was declared in Wuhan, China, on 17 November 2019 [6]. Then the World Health Organization (WHO) declared it a global pandemic on 11 March 2020, because of its rapid spreading [7]. Thus, COVID-19 started and was followed by subsequent global outbreaks for months. The WHO recorded over 218 million reported cases of Coronavirus and approximately 4.5 million deaths globally until 1 September 2021 [8]. Because of the disease’s rapid dissemination at the beginning of the pandemic, governments worldwide were forced to impose strict measures to stop or decrease its spread, such as total or partial lockdowns, quarantine, and social distancing [9,10,11,12].

In Jordan, parallel with most countries worldwide, the government quickly restrained the spread of the virus due to this outbreak. Complete lockdowns began on 21 March 2020, for two weeks, and partial lockdowns were implemented until 1 September 2021, closing the non-essential public places. In addition, telework and distance learning was initiated, delivery services such as delivering drugs to chronically ill patients were provided, and during the night and the lockdown, cities were sanitized as part of the National Disinfection Program [11]. 

During the quarantine worldwide, uncertainty about the future led work owners to reduce the number of workers or the wages because of the spread of this virus. Moreover, with the work shortages and salaries reductions, healthy nutrition accessibility and affordability were compromised, causing people to adopt more palatable, cheaper, and potentially unhealthy choices, affecting their diet consistency [13,14,15]. Several studies noted that an unhealthy diet and the side effects of the quarantine on movement have negatively impacted people and their immune status [16]. People’s psychological status was also affected because of the long time they spent in their houses, being prevented them from going outdoors. Because of these reasons, people focused on their daily needs, such as cooking, eating, and sleeping. Moreover, people increased their laziness, decreased their amount of exercise, and adopted poor eating habits. Several researchers have shown the harmful effects of negative eating habits such as elevated calorie intake, more regular snacking, decreased fresh fruit and vegetable consumption, and weight gain during the lockdown [15,16].

A study conducted in the UK and Scotland [17] attempted to identify the effect of lifestyle restrictions on mental health. It found that the changes in diet, quality of sleep, and physical activity negatively affected people’s moods and health behaviors in the lockdown. Another study conducted in Australia found a significant effect of the lockdown on social connectedness, relationships, financial stress, health-promoting behaviors, and emotional well-being [18]. A study conducted in Cyprus found that COVID-19 lockdown affected all lifestyle aspects: diet, stress, socialization, and physical activity [19]. An online cross-sectional survey conducted during the social lockdown in the United Kingdom found that lifestyle behaviors associated with weight gain are likely to have been affected by the COVID-19 crisis. Successful weight control was not possible with poor diet and binge eating habits. Therefore, people with mental health and obesity problems could be at higher risk [20]. Another study also discussed the tendency of eating ready food instead of homemade meals and their effect on the weight and BMI of subjects [21].

In Jordan, restaurants and shops were closed during the full quarantine for twenty-seven days from 15 March to 12 April 2020, and home delivery of fast food, meals, and daily requirements were highly restricted. The lockdown has impacted the eating habits of people during the pandemic. Most households depended on home cooking at least for their main meals because of the limited access to fast food, food delivering, and staying at home. Recent studies have discussed the effect of diet and lifestyle on the health of the Jordanian population. Of these studies, one [22] discussed the effect of sedentary hours, homemade food, and fast food on obesity and body mass index (BMI). The increase in sedentary hours, lack of adequate daily exercise, and fast food and snacking habits increased obesity in the Jordanian population. 

In order to collect better quality data, a reliable and valid questionnaire was constructed. Validity and reliability are two fundamental elements used in evaluating questionnaires. Validity refers to the appropriateness, significance, and usefulness of a measure for a specific purpose. In addition, it refers to the extent to which the measures are useful predictors of essential outcomes [23]. Reliability is concerned with the ability of a questionnaire to measure consistently. The reliability of a questionnaire does not depend on its validity, and a questionnaire cannot be valid unless it is reliable [24].

To ensure that the questionnaire is reliable, we should provide a measure for internal consistency. It must be noted that internal consistency should be determined before examining the survey to ensure validity. Cronbach Alpha, which is considered one of the most widespread reliability measure methods, was developed by Lee Cronbach in 1951 to indicate internal consistency [24]. It is associated with the inter-relatedness of questions, which indicates that items in the test measure have the same construct. The alpha value is expressed as a number range between (0 and 1) depending on the test’s nature. The value of alpha increases if the items in a test are correlated, which means that items are more strongly interrelated. Cronbach’s alpha equal to zero indicates no internal consistency, whereas alpha equal to one reflects perfect internal consistency. Still, it does not mean that if we have a high alpha value, the test always has a high degree of internal consistency, because sometimes it indicates that some items may be redundant or may be affected by the length of the questionnaire. The alpha value decreases if the test length is shorter and increases as the number of items and variability of each item increases [25]. Practically, Cronbach’s alpha of at least 0.70 has been suggested to indicate adequate internal consistency.

Validity means to “measure what is intended to be measured” [26]. It is essential to realize that any measurement technique measures what it is designed to measure. It is much easier to assess with the help of principles component analysis (PCA). PCA is a dimensionality—a statistical reduction technique. It was initially developed to enhance the understanding of questionnaires composed of a large number of correlated variables. It is achieved by transforming many possibly correlated variables into a smaller number called ‘principal components’ while retaining the variation present in the data set. Thus, a smaller data set of uncorrelated variables is easier to understand, realize, visualize, and use in further analysis than a more significant set [27,28].

Some researchers [24,25,26,27,28,29] are interested in measuring the reliability and validity of their questionnaires to achieve good results, but sometimes they may hesitate to use the alpha method to test reliability. Because alpha is affected by the number of items, increasing the number of items could indicate a high similarity, but the correlation does not change. Moreover, it requires the question’s covariances to be equivalent, implying they have at least one common factor. Likewise, the PCA technique to test the validation of a survey has a drawback that may affect its application. If the covariance of the data obtained is difficult to evaluate accurately, it could affect the accuracy of the results obtained later. 

The COVID-19 pandemic restriction implementation has been evaluated in a number of countries in terms of its impact on diet and lifestyle. As each country implements different pandemic restrictions depending on the severity of the virus transmission, there is an expectation that it will affect lifestyle behaviors and health differently depending on where they were implemented. A specific study is thus needed for Jordan. 

This study examined changes in Jordanian diet and lifestyle habits during the COVID-19 pandemic as well as possible associations with changes in body mass index using an online questionnaire. In order to assess lifestyle-related behaviors, this questionnaire asked short, concise, straightforward, scientifically structured, and easy-to-use questions for the Jordanian adult population.

## 2. Materials and Methods

### 2.1. Subjects

The questionnaire population consisted of Jordanian people from different social strata above 18. We received 467 responses distributed as follows: 297 (64%) responses from females and 170 (36%) from males. The population age was between 18 and 103 years old and the mean was 33.9 years (SD 13.1). The mean of the respondents’ height was 168.2 cm (SD 9.4). In addition, the mean for the respondents’ weight was 73.4 kg (SD 18.3). A total of four hundred respondents (86%) lived within a small family formed by mother and father and their children only, called the nuclear family. The other 67 (14%) respondents lived as an extended family consisting of grandparents, uncles, parents, and children.

### 2.2. Research Tools and Data Collection

The questionnaire was prepared previously in research “A short questionnaire to assess changes in lifestyle-related behavior during COVID-19 pandemic” [30]. In the current study, this questionnaire was applied to the Jordanian society with an adult population of 6,000,000 after translating the questionnaire from English into Arabic. Using the Raosoft software, we calculated the necessary number of responses, which was 385 or more, to have a confidence level of 95%. The real value is within ±5% of the measured and surveyed values. The 22-item questionnaire was completed by Jordanian people aged 18 years and above who could read, write, and respond to an online web-based questionnaire. Responses under each item consist of significantly increased, slightly increased, grossly similar, slightly decreased and significantly decreased. In scoring, five points were assigned to answer “significantly increased”, and one point was assigned to answer “significantly decreased”. In addition, participants were recruited in different demographic strata such as age, gender, and socio-economic status. Questionnaires were completed online by using Google forms. The online data were collected in August 2021. In the beginning, a sample of 71 respondents’ data was collected to validate the questionnaire and check its reliability. Then, a total of 467 responses was received. All the candidates completed the questionnaire by themselves, and there were no missed answers in the responses. 

### 2.3. Statistical Analysis

#### 2.3.1. Construct Validity and Reliability

The Cronbach’s α coefficients for the questions were calculated. The α coefficient reflects the degree of internal consistency directly. The validity of a questionnaire was established by construct-related evidence. Items were subjected to a principal components analysis.

#### 2.3.2. Data Analysis

Descriptive statistics were used to analyze the sociodemographic variables by counts and percentages for discrete variables and mean and SD for continuous variables. Afterward, Q-Q plots and O’Brien test were used to assess normality and unequal variances for the BMI difference variable, respectively. Because no violations were found, ANOVA was used to test the means of BMI difference. Then if the *p*-value was significant for the ANOVA, Tukey–Kramer HSD was applied to study pairwise comparisons and find the differences. On the other hand, Q-Q plots and O’Brien test were used for the age variable. A violation was found for normality assumption. Therefore, log transformation was applied before the ANOVA model was utilized. Moreover, the Chi-square test was used to study the effect of demographic variables on the respondents’ choices for questions from question 10 to question 31. The correlation between questions was assessed using Pearson’s correlation coefficient.

All analyses were performed using JMP software (SAS Institute Inc., Cary, NC, USA) with confidence interval α = 0.05. 

## 3. Results

### 3.1. Reliability of the Questionnaire 

Cronbach α is calculated to know the internal consistency. All Cronbach α values for the questionnaire are greater than 0.7, as shown in Table 1. It is considered an accurate estimate for reliability because the values of 0.7 or 0.8 are considered an acceptable high value [31,32,33].

### 3.2. Validation of the Questionnaire 

The questionnaire’s factor structure was analyzed using the principal components analysis. A total of two tests and two *p*-values were used. The first test $\chi_{231}^{2}$ = 645.8, *p*-value ˂ 0.0001, which is a significant value, so the $H_{0}$ was rejected, which indicated that there was at least a common factor between the questions. The second test $\chi_{131}^{2}$ = 152.7, *p*-value = 0.09. So $H_{a}$ was rejected. That indicated that no more than five factors were needed to describe the principal component. The item loading of 0.40 or more under these five factors was considered. Studying the factor loadings highlights that each factor has an explanation except for factor five. As factor five had only two items, one of them had a negative sign; this indicates that question number eleven had the negative sign, and is excluded from the computation because it had a negative effect on the factors.

We named the first factor “bad dietary habits” because all the questions discuss changes in negative eating habits during the pandemic. The second factor was called “health social awareness,” the third factor “doing activities” and the fourth factor “bad dietary habits.” As seen in Table 2, factor 1 included eight items, factor two included four items, factor three included three items, and factor four included two items. These four factors accounted for 43.5% of the variance. Factor 1 accounted for 18.1% of the total variance, factor 2 accounted for 10.9%, factor 3 accounted for 7.4%, and factor 4 accounted for 7.1%.

The results of the principal component analyses with subsequent Varimax rotation are detailed in Table 2:

**Table 2:** Principal component analyses result with subsequent Varimax rotation.

Question Number	Factor 1 (18.1%)	Factor 2 (10.9%)	Factor 3 (7.4%)	Factor 4 (7.1%)	Factor 5 (5.7%)
Q 13	0.8191				
Q 20	0.7740				
Q 14	0.7411				
Q 22	0.6608				
Q 18	0.6267				
Q 19	0.6053				
Q 10	0.5713				
Q 17	0.5294				
Q 26		0.7594			
Q 25		0.7459			
Q 23		0.6855			
Q 24		0.4218			
Q 28			0.61757		
Q 29			0.5883		
Q 21			0.5323		
Q 16				0.7288	
Q 15				0.7130	
Q 12					0.6718
Q 11					−0.5038
Q 27					
Q 30					

### 3.3. Demographics Summary

Because reliability was studied and it showed good internal consistency, and the validation proved that the questionnaire could be used successively, we proceeded with the analyses.

All responses were analyzed to determine the effects of each variable on the BMI difference and how each variable interacted with others.

**Figure 1:**
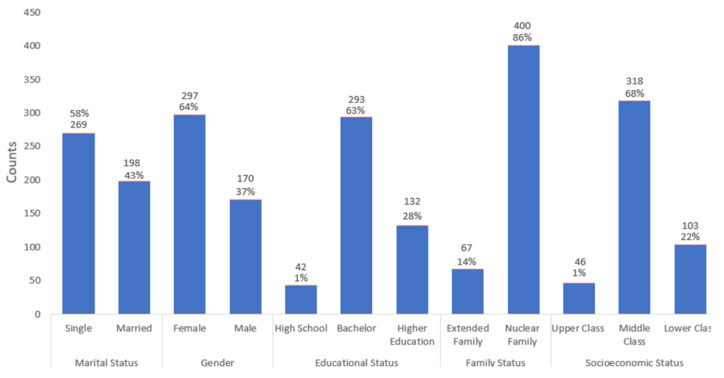
Percentages and counts of respondents across demographic variables.

The following chart (Figure 1) contains a summary of demographic variables. 

**Figure 2:**
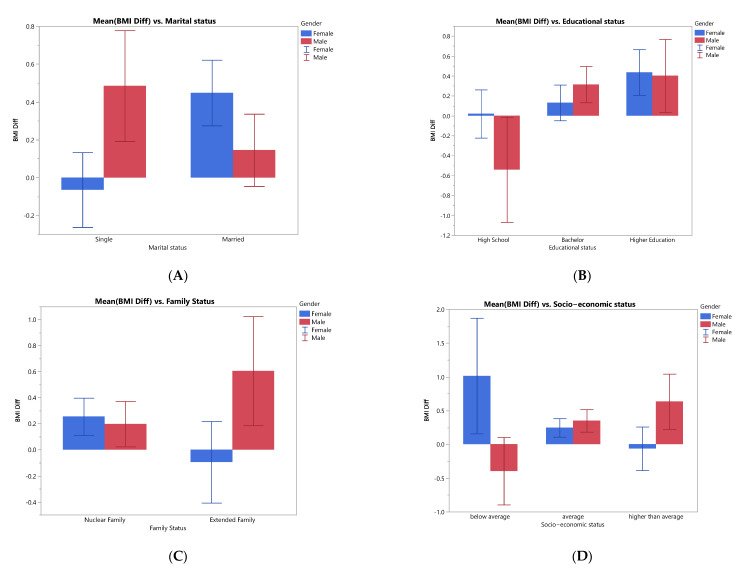
(**A**) Summary of BMI difference values for the respondents regarding Marital Status by gender, (**B**) Summary of BMI difference values for the respondents regarding educational status by gender, (**C**) Summary of BMI difference values for the respondents regarding family status by gender, (**D**) Summary of BMI difference values for the respondents regarding socio-economic Status by gender.

The following figures (Figure 2A–D) summarize the BMI changes for demographic data for gender variable before and after the lockdowns. The first figure shows a difference between single males and single females; single males increased their BMI, in contrast with single females’ BMI, where their BMI decreased during the COVID-19 pandemic. On the other hand, married females showed an increase in BMI over married males. 

In the second figure, there has been a decrease in BMI for males from the high school stratum compared to males from the bachelor and higher educational strata, where there has been an increase in BMI. Nevertheless, males’ BMI increment in the bachelor stratum was higher than female increment, but the difference was not as large in the higher educational stratum.

In the third figure, males and females from the nuclear family level clearly increased their BMI during the pandemic, where males in the extended family level showed an increase in their BMI while females from the same category showed a slight decrease in their BMI. 

Lastly, the fourth figure for the socio-economic variable shows that males from below-average stratum decreased their BMI compared to males from the two other strata who increased their BMI. On the other hand, the females showed an increase in the lower and average strata while showing stability in the higher stratum.

Table 3 shows percentages for “slightly increased” and “significantly increased” levels for the questions that showed high response rates for these levels.

For each question regarding the BMI difference, an ANOVA was used to compare levels. Needed assumptions were tested. Unequal variances for each variable were examined, and they were found to be insignificant for most questions. The highest and lowest SD ratios for the questions with significant unequal variance tests were calculated. All ratios were around two, which is acceptable for assuming equal variances. Moreover, the normality of the BMI difference was tested by using a Q-Q plot per group for each variable, and no violation for the normality assumption to use ANOVA was found. As a result, twelve questions having significant means differences were found. *p*-values are listed in Table 4. 

To find where the differences are, Tukey–Kramer HSD was used. All significant pairwise comparisons are listed in Table 5. The pairwise comparisons for questions 25 and 27 are not significant, although their *p*-values for their models are significant.

Moreover, for the other factors (gender, marital status, educational status, family status, and socio-economic status), the significance of their effect over the choices of each question was studied using the usual Chi-square test. Moreover, the *p*-values for questions with significant effects are stated in Table 6. 

In the following part of the study, the percentages for each choice among those with significant *p*-values were studied.

A total of three significant questions associated with gender variable were found. In the three questions, females showed a greater increase in the percentage than males; in question sixteen, females (18.5%) showed more increase in consuming healthy food than males (8%). Likewise, in question twenty-two, females (36.7%) increased cooking new and traditional meals more so than males (18.2%). In question twenty-eight, females (32.3%) increased the number of household chores more so than males (17.7%).

A total of eight significant questions were associated with the marital status variable. Percentages for each question are shown in Table 7. In question thirteen, single people (51%) increased the amount of consuming snacks between the meals less than married people (61%). Moreover, in question thirty, there is a significant difference in the increase in hours of sleep, where single people (59%) increased the amount of sleep more so than married people (43.4%).

A total of six significant questions were associated with the educational status variable. Percentages for each question are shown in Table 8. A variation between the high school, bachelor and highly educated people in these questions was noted. For example, in question thirteen, high school students increased their snacking by only 40%, whereas the bachelor and highly educated people by 57% and 58%, respectively. 

The family status variable was significantly associated with two questions. More than half of nuclear families (59%) snack between meals, compared to just 35% of extended families. Furthermore, 50% of nuclear families ate unhealthy foods when they were bored, stressed, or upset, compared to only 36% of extended families. A significant association was found between socio-economic status and twelve questions. The percentages for each question are shown in Table 9. The habits of people in the higher income level increased for some questions. For example, the increment for people with a higher-than-average income who kept a balanced diet (54%) was greater than average and below-average levels of income(40% and 24%, respectively). For other questions, people with average income reported higher increments than the other two levels. For example, the increment for average level intake of fruits and vegetables (49%) was greater than the higher-than-average and below-average levels (41% and 33%, respectively). 

Our study identified several significant correlations between the questions using the Pearson correlation. It has been noted that the good habits questions were positively correlated, as well as the bad habits questions. For example, there is a significant correlation between snack intake in question fourteen and the number of snacks eaten between meals in question thirteen because the correlation value exceeded 0.71. Based on the correlation of 0.58 between questions fifteen and sixteen, most people who increased their intake of fruits and vegetables also increased their consumption of a balanced diet. As indicated by the positive correlations between question twenty-two and questions fourteen, eighteen and twenty, when people were bored, stressed, or upset, they tended to eat more fried food, sweets, candies, and chocolate, and snack between meals. The correlation between question twenty-four and question twenty-five suggested that most people increased their intake of nutritional supplements when supported by family and friends to eat healthily. The other correlation values are shown in Table 10.

The association between sleep patterns and age has been studied. The respondents who increased their sleeping hours tended to have a younger mean of age for all significant pairwise comparisons. However, the age data did not follow the normality assumptions, so the data were transformed using the Log transform before the analyses. For more information about Log transform, see [34]. 

**Figure 3:**
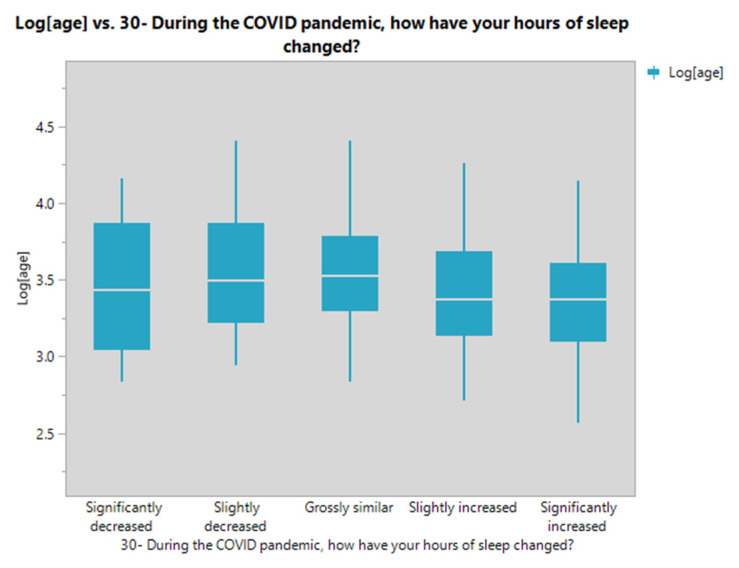
Plot for the log of age by sleeping patterns.

The significant pairwise comparisons from the Tukey–Kramer HSD test were found, and the differences were back-transformed (Table 11). According to Figure 3, the log age mean for the slightly decreased level is higher than that for the significantly increased level. The log age mean for the grossly similar level is higher than the slightly and significantly increased levels. 

## 4. Discussion

According to the propagation of COVID-19, many countries worldwide performed total and partial lockdowns to prevent the spreading of the virus. Those lockdowns affected people’s psychological, dietary, health, and social behaviors [35,36,37,38].

Using a 22-item web-based questionnaire, this study sought to discover the impact of COVID-19 lockdown on Jordanian adults during and after the lockdown.

First, the questionnaire’s validity and reliability were checked; the questionnaire had a good internal consistency using Cronbach α. Each question had an α value between 0.7 and 0.75. That showed a good level of reliability.

Moreover, the validity of the questionnaire was studied using principal component analysis. Questions were distributed to five factors. The factors labeled are bad dietary habits, health, social awareness, activities, and consuming healthy food. The last factor was omitted because it consists of two questions, one of which has a negative sign that was excluded from the computation. The principal component analyses showed acceptable validity. 

Using ANOVA, the effects of the questions on the difference in BMI were then tested. A total of twelve significant questions were identified (12, 13, 14, 17, 18, 19, 20, 22, 25, 26, 27, and 29). After pairwise comparisons, in question twenty-six, which measures a healthy habit (During the COVID-19 pandemic, how has your interest in learning healthy eating tips from the media (newspaper articles, magazines, blogs, videos, T.V. shows, and text messages) changed?), the decline of this habit was associated with a higher BMI difference. For the other questions describing bad dietary habits, there has been a higher BMI difference mean for those who have increased these habits than those who have decreased them. These results suggest that a decrease in bad dietary habits is related to decreased BMI difference. This is expected and logical, so the questionnaire’s integrity is supported. Therefore, enhancing people’s awareness towards increased intake of healthy food and adopting good eating habits is advised. Other studies have reached the same results [39,40,41].

Furthermore, factors such as gender, marital status, education level, family status, and socio-economic status were studied for their effects on the choices made for questions. We found three significant questions relating to gender, eight to marital status, six to educational status, two to family status, and twelve to socio-economic status. For the gender factor, our results showed that females generally have a healthier lifestyle than males by eating healthier and being more active. Other studies found females in general to have a healthy lifestyle and to have healthier eating and drinking habits than males. Females were also found to have greater motivation toward being active and males to be more likely to smoke and be overweight [32,38,42,43]. 

Family Status is one of the main factors in the questionnaire, which has a significant effect. Extended families demonstrated more commitment to not eating snacks between meals, increasing by only 20.9%, whereas nuclear families increased their snack consumption by 26.25%. Several studies illustrated that living in extended families encourages people to live healthier than living in nuclear families [43,44,45]. People in extended families were more likely to favor vegetable-based meals. On the other hand, people in nuclear families enjoyed snacks more. Oyen et al. [45] showed that individuals living with a spouse or in a nuclear family had a higher probability of mental ill-health in the absence of people showing concern for their well-being in comparison to extended families.

Concerning the educational status, in six questions, there was a difference between those with high school education and bachelor’s degrees and those from highly educated backgrounds. Those with higher education were more committed to implementing good habits than those with bachelor’s degrees, as well as those with high school diplomas. Some studies showed that there is a link between a lower educational level and a higher risk of health issues, which is related to a lower knowledge of health information and awareness [46]. In some studies, there was a favorable relationship between the parents’ higher degree of academic education and the frequency of consuming quick meals and snack consumption [47,48,49,50]. 

A significant association was found between socio-economic status and twelve questions. For some questions, people with higher-than-average income increased their habits. For example, the increment for their intake of a balanced diet was higher than for individuals with average or below-average levels. With regard to other questions, average income individuals have more habit increments than individuals with below-average or higher-than-average incomes. For people with below-average income, their financial capability may have prevented them from increasing their bad eating habits, as expected.

Eisinga et al. [32] found that people with lower income and education have less healthy dietary habits due to their preference for familiar food and price. They regarded health as the least important factor in their food purchase decisions. The dietary habits of people in the higher education and average salary stratum did not change much, in contrast to those with low levels of education and low salaries, who showed a significant increase in stress and anxiety.

The age mean differences between sleeping patterns levels were explored using an ANOVA. Results showed that groups with increased sleeping hours tended to have a younger mean of age. A young person’s boredom and delayed sleep phase (going to bed and waking up) may account for this increment. A recent study supports our findings that older participants had shorter sleep duration, along with a suggestion that maintaining a schedule could be a protective factor in addressing sleep issues [51]. 

In Jordan, the COVID-19 crisis might have influenced eating habits, physical activity, and weight-related lifestyle behaviors, and Jordanians’ habits may have been significantly altered as compared to what they used to be. Although certain positive behaviors increased, such as consuming home-cooked meals, the quality and the quantity of the food were compromised. Carbohydrates and sugar consumption plays a significant role in eating healthy. The concept of eating healthy varies and may be misleading to some individuals depending on their background and educational level. 

Consequently, public health officials must focus on nutrition awareness by suggesting healthy food choices and nutritious substitutes throughout pandemics, especially in lockdown conditions. It is strongly recommended that individuals improve their physical activity, have enough sleep, and avoid eating energy-dense ‘junk’ food, leading to weight gain, supine immunity system, and COVID-19 vulnerability [52].

## 5. Conclusions

A study of Jordanian adults was conducted to determine how the COVID-19 lockdown affected their eating habits, their physical activity, and weight gain. Results showed that males were more likely to gain weight than females. In addition, the questionnaire revealed that the family environment was significant. Nuclear families increased snacking, whereas extended families increased vegetable consumption. People with high socio-economic status tend to make healthier dietary choices than those with low socio-economic status.

The COVID-19 epidemic has altered Jordanians’ eating habits. Although positive behaviors have grown, such as eating home-cooked meals, food quality and quantity have been compromised.

To this end, public health officials need to promote nutrition awareness. Physical activity and having enough sleep are recommended, as well as avoiding eating ‘junk’ food.

## Figures and Tables

**Table 1:** Cronbach α values for the questionnaire questions.

Question Number	Question	Alpha
Q 10	During the COVID-19 pandemic, how has your weight changed?	0.7277
Q 11	Have you ever had COVID-19?	0.7530
Q 12	During the COVID-19 pandemic, how has your probability of skipping one of the main meals (breakfast, lunch, or dinner) changed?	0.7296
Q 13	During the COVID-19 pandemic, how has your habit of snacking between meals changed?	0.7046
Q 14	During the COVID-19 pandemic, how has your quantity and portions of meals and snacks changed?	0.7152
Q 15	During the COVID-19 pandemic, how has your daily intake of fruits and vegetables changed?	0.7387
Q 16	During the COVID-19 pandemic, how has your intake of a balanced diet (including healthy ingredients such as whole wheat, pulses, legumes, eggs, nuts, fruits, and vegetables) changed?	0.7515
Q 17	During the COVID-19 pandemic, how has your consumption of junk food and fast food changed?	0.7277
Q 18	During the COVID-19 pandemic, how has your consumption of fried food changed?	0.7247
Q 19	During the COVID-19 pandemic, how has your intake of sugar-sweetened beverages (carbonated soft drinks and sugar-sweetened juices) changed?	0.7221
Q 20	During the COVID-19 pandemic, how has your consumption of sweets, candies, and chocolate changed?	0.7048
Q 21	During the COVID-19 pandemic, how has your participation in cooking new or traditional recipes changed?	0.7392
Q 22	During the COVID-19 pandemic, how has your consumption of unhealthy food when you are bored, stressed, or upset changed?	0.7212
Q 23	During the COVID-19 pandemic, how has your intake of immunity-boosting foods (lemon, garlic, turmeric, green leafy vegetables, and citrus fruits) in the diet changed?	0.7403
Q 24	During the COVID-19 pandemic, how has your intake of nutrition supplements to boost immunity changed?	0.7413
Q 25	During the COVID-19 pandemic, how has the support of your family and friends in eating healthy changed?	0.7499
Q 26	During the COVID-19 pandemic, how has your interest in learning healthy eating tips from the media (newspaper articles, magazines, blogs, videos, T.V. shows, and text messages) changed?	0.7471
Q 27	During the COVID-19 pandemic, how has your participation in aerobic exercise changed?	0.7416
Q 28	During the COVID-19 pandemic, how has your participation in leisure and household chores changed?	0.7407
Q 29	During the COVID-19 pandemic, how has your sitting and screen time changed?	0.7260
Q 30	During the COVID-19 pandemic, how have your hours of sleep changed?	0.7405
Q 31	During the COVID-19 pandemic, how have your stress and anxiety levels changed?	0.7395

**Table 3:** Percentages of participants who showed an increased behavior according to questions (slightly increase and significantly increase).

Question Number	Question	Slightly Increase	Significantly Increase
Q13	During the COVID-19 pandemic, how has your habit of snacking between meals changed?	32%	25%
Q14	During the COVID-19 pandemic, how has your quantity and portions of meals and snacks changed?	30%	20%
Q21	During the COVID-19 pandemic, how has your participation in cooking new and traditional recipes changed?	23%	30%
Q23	During the COVID-19 pandemic, how has your intake of immunity-boosting foods (lemon, turmeric, garlic, green leafy vegetables, and citrus fruits) in the diet changed?	36%	26%
Q24	During the COVID-19 pandemic, how has your intake of nutrition supplements to boost immunity changed?	33%	23%
Q26	During the COVID-19 pandemic, how has your interest in learning healthy eating tips from the media (newspaper articles, magazines, blogs, videos, T.V. shows, and text messages) changed?	35%	19%
Q28	During the COVID-19 pandemic, how has your participation in leisure and household chores changed?	31%	26%
Q29	During the COVID-19 pandemic, how has your sitting and screen time changed?	24%	35%
Q30	During the COVID-19 pandemic, how have your hours of sleep changed?	25%	25%
Q31	During the COVID-19 pandemic, how have your stress and anxiety levels changed?	34%	40%

**Table 4:** The *p*-value for each question with significant means differences.

Question Number	Question	*p*-Value
Q 12	During the COVID-19 pandemic, how has your probability of skipping one of the main meals (breakfast, lunch, and dinner) changed?	0.0002
Q 13	During the COVID-19 pandemic, how has your habit of snacking between meals changed?	<0.0001
Q 14	During the COVID-19 pandemic, how has your quantity and portions of meals and snacks changed?	<0.0001
Q 17	During the COVID-19 pandemic, how has your consumption of junk food and fast food changed?	<0.0001
Q 18	During the COVID-19 pandemic, how has your consumption of fried food changed?	<0.0001
Q 19	During the COVID-19 pandemic, how has your intake of sugar-sweetened beverages (carbonated soft drinks and sugar-sweetened juices) changed?	<0.0001
Q 20	During the COVID-19 pandemic, how has your consumption of sweets, candies, and chocolate changed?	<0.0001
Q 22	During the COVID-19 pandemic, how has your consumption of unhealthy food when you are bored, stressed, upset changed?	<0.0001
Q 25	During the COVID-19 pandemic, how has the support of your family and friends in eating healthy changed?	0.0316
Q 26	During the COVID-19 pandemic, how has your interest in learning healthy eating tips from the media (newspaper articles, magazines, blogs, videos, T.V. shows, and text messages) changed?	0.0182
Q 27	During the COVID-19 pandemic, how has your participation in aerobic exercise changed?	0.0275
Q 29	During the COVID-19 pandemic, how has your sitting and screen time changed?	<0.0001

**Table 5:** The results of Tukey–Kramer HSD for significant pairwise comparisons.

Level (Mean ± SD)	Level (Mean ± SD)	Difference	95% CI	*p*-Value
Q.12 During the COVID-19 pandemic, how has your probability of skipping one of the main meals (breakfast, lunch, and dinner) changed?				
5 (0.99 ± 3.01)	2 (−0.31 ± 1.50)	1.30	(0.27, 2.31)	0.0051
5 (0.99 ± 3.01)	1 (−0.25 ± 3.35)	1.24	(0.11, 2.37)	0.0227
5 (0.99 ± 3.01)	3 (−0.07 ± 1.66)	1.06	(0.28, 1.84)	0.0020
Q.13 During the COVID-19 pandemic, how has your habit of snacking between meals changed?				
5 (0.99 ± 2.99)	1 (−1.49 ± 2.62)	2.48	(1.21, 3.74)	<0.0001
4 (0.55 ± 1.57)	1 (−1.49 ± 2.62)	2.04	(0.80, 3.28)	<0.0001
5 (0.99 ± 2.99)	2 (−0.70 ± 1.95)	1.70	(0.67, 2.72)	<0.0001
3 (−0.2 ± 1.51)	1 (−1.49 ± 2.62)	1.29	(0.03, 2.54)	0.0413
4 (0.55 ± 1.57)	2 (−0.70 ± 1.95)	1.26	(0.26, 2.25)	0.0055
5 (0.99 ± 2.99)	3 (−0.20 ± 1.51)	1.20	(0.47, 1.92)	<0.0001
4 (0.55 ± 1.57)	3 (−0.20 ± 1.51)	0.75	(0.06, 1.44)	0.0242
Q.14 During the COVID-19 pandemic, how has your quantity and portions of meals and snacks changed?				
5 (1.11 ± 2.97)	1 (−1.55 ± 2.42)	2.66	(1.36, 3.97)	<0.0001
5(1.11 ± 2.97)	2 (−1.35 ± 2.62)	2.47	(1.47, 3.46)	<0.0001
4 (0.71 ± 1.72)	1 (−1.55 ± 2.42)	2.26	(1.00, 3.53)	<0.0001
4 (0.71 ± 1.72)	2 (−1.35 ± 2.62)	2.06	(1.13, 3.00)	<0.0001
3 (0.002 ± 1.48)	1 (−1.55 ± 2.42)	1.55	(0.30, 2.81)	0.0067
3 (0.002 ± 1.48)	2 (−1.35 ± 2.62)	1.36	(0.43, 2.28)	0.0007
5 (1.11 ± 2.97)	3 (0.002 ± 1.48)	1.36	(0.38, 1.84)	0.0004
4 (0.71 ± 1.72)	3 (0.002 ± 1.48)	1.11	(0.06, 1.36)	0.0240
Q.17 During the COVID-19 pandemic, how has your consumption of junk food and fast food changed?				
5 (1.74 ± 2.41)	1 (−0.56 ± 2.66)	2.30	(1.41, 3.19)	<0.0001
5 (1.74 ± 2.41)	4 (0.09 ± 1.95)	1.65	(0.71, 2.60)	<0.0001
5 (1.74 ± 2.41)	2 (0.12 ± 1.93)	1.61	(0.62, 2.61)	0.0001
5(1.74 ± 2.41)	3 (0.33 ± 1.45)	1.41	(0.54, 2.28)	0.0001
3 (0.33 ± 1.45)	1 (−0.56 ± 2.66)	0.89	(0.16, 1.62)	0.0084
Q.18 During the COVID-19 pandemic, how has your consumption of fried food changed?				
5 (1.39± 2.59)	1 (−1.14 ± 2.20)	2.53	(1.32, 3.74)	<0.0001
4 (0.89 ± 2.22)	1(−1.14 ± 2.20)	2.02	(0.91, 3.14)	<0.0001
1(−1.14 ± 2.2)	2 (−0.44 ± 2.59)	1.83	(0.79, 2.87)	<0.0001
4 (0.89 ± 2.22)	2 (−0.44 ± 2.59)	1.32	(0.39, 2.26)	0.0011
5 (1.39± 2.59)	3 (0.08 ± 1.69)	1.32	(0.46, 2.18)	0.0003
3 (0.08 ± 1.69)	1(−1.14 ± 2.20)	1.21	(0.20, 2.23)	0.0101
4 (0.89 ± 2.22)	3 (0.08 ± 1.69)	0.81	(0.08, 1.54)	0.0207
Q.19 During the COVID-19 pandemic, how has your intake of sugar-sweetened beverages (carbonated soft drinks and sugar-sweetened juices) changed?				
5 (1.18± 2.52)	1 (−0.71± 2.89)	1.89	(0.90, 2.88)	<0.0001
5 (1.18± 2.52)	2 (−0.57± 1.65)	1.75	(0.73, 2.76)	<0.0001
4 (0.87± 2.14)	1 (−0.71± 2.89)	1.57	(0.62, 2.52)	<0.0001
4 (0.87± 2.14)	2 (−0.57± 1.65)	1.43	(0.45, 2.41)	0.0007
5 (1.18± 2.52)	3 (0.18± 1.71)	1.00	(0.19, 1.81)	0.0071
3 (0.18± 1.71)	1 (−0.71± 2.89)	0.89	(0.07, 1.60)	0.0269
Q.20 During the COVID-19 pandemic, how has your consumption of sweets, candies, and chocolate changed?				
5 (1.13 ± 2.44)	1 (−0.96 ± 1.97)	2.0835	(1.02, 3.15)	<0.0001
5 (1.13 ± 2.44)	2 (−0.62 ± 3.05)	1.75	(0.76, 2.73)	<0.0001
4 (0.57 ± 1.85)	1 (−0.96 ± 1.97)	1.53	(0.49, 2.56)	0.0006
5 (1.13 ± 2.44)	3 (−0.10 ± 1.64)	1.22	(0.48, 2.97)	<0.0001
4 (0.57 ± 1.85)	2 (−0.62 ± 3.05)	1.19	(0.23, 2.14)	0.0063
Q.22 During the COVID-19 pandemic, how has your consumption of unhealthy food changed when you are bored or stressed, or upset?				
5 (1.19 ± 2.88)	1 (−0.81 ± 2.13)	2.00	(0.80, 3.20)	<0.0001
5 (1.19 ± 2.88)	2 (−0.26 ± 1.78)	1.45	(0.34, 2.56)	0.0035
5 (1.19 ± 2.88)	3 (−0.24 ± 1.58)	1.43	(0.67, 2.19)	<0.0001
4 (0.59 ± 2.24)	1 (−0.81 ± 2.13)	1.39	(0.25, 2.53)	0.0079
4 (0.59 ± 2.24)	3 (−0.24 ± 1.58)	0.82	(0.16, 1.49)	0.0070
Q.26 During the COVID-19 pandemic, how has your interest in learning healthy eating tips from the media (newspaper articles, magazines, blogs, videos, T.V. shows, and text messages) changed?				
2 (1.67 ± 1.8)	5 (−0.28 ± 3.04)	1.96	(0.24, 3.67)	0.0163
Q.29 During the COVID-19 pandemic, how has your sitting and screen time changed?				
5 (0.65 ± 2.31)	1 (−1.71 ± 4.65)	2.36	(0.94, 3.79)	<0.0001
2 (0.54 ± 1.58)	1 (−1.71 ± 4.65)	2.26	(0.35, 4.17)	0.0114
4 (0.34 ± 1.76)	1 (−1.71 ± 4.65)	2.06	(0.60, 3.52)	0.0012
3 (−0.11 ± 1.79)	1 (−1.71 ± 4.65)	1.60	(0.17, 3.04)	0.0195
5 (0.65 ± 2.31)	3 (−0.11 ± 1.79)	0.76	(0.10, 1.42)	0.0149

**Table 6:** The *p*-values of the significant questions concerning the demographics variables.

Question Number		*p*-Value
Gender		
Q.16	During the COVID-19 pandemic, how has your intake of a balanced diet (including healthy ingredients such as whole wheat, pulses, legumes, eggs, nuts, fruits, and vegetables) changed?	0.0129
Q. 21	During the COVID-19 pandemic, how has your participation in cooking new and traditional recipes changed?	<0.0001
Q. 28	During the COVID-19 pandemic, how has your participation in leisure and household chores changed?	0.0001
Marital status		
Q.12	During the COVID-19 pandemic, how has your probability of skipping one of the main meals (breakfast, lunch, and dinner) changed?	0.0049
Q.13	During the COVID-19 pandemic, how has your habit of snacking between meals changed?	0.0097
Q.14	During the COVID-19 pandemic, how has your quantity and portions of meals and snacks changed?	0.0478
Q.16	During the COVID-19 pandemic, how has your intake of a balanced diet (including healthy ingredients such as whole wheat, pulses, legumes, eggs, nuts, fruits, and vegetables) changed?	0.0410
Q.21	During the COVID-19 pandemic, how has your participation in cooking new and traditional recipes changed?	0.0299
Q.23	During the COVID-19 pandemic, how has your intake of immunity-boosting foods (lemon, garlic, turmeric, green leafy vegetables, and citrus fruits) in the diet changed?	0.0191
Q.24	During the COVID-19 pandemic, how has your intake of nutrition supplements to boost immunity changed?	0.0037
Q.30	During the COVID-19 pandemic, how have your hours of sleep changed?	0.0007
Educational status		
Q. 13	During the COVID-19 pandemic, how has your habit of snacking between meals changed?	0.0213
Q. 16	During the COVID-19 pandemic, how has your intake of a balanced diet (including healthy ingredients such as whole wheat, pulses, legumes, eggs, nuts, fruits, and vegetables) changed?	0.0007
Q. 24	During the COVID-19 pandemic, how has your intake of nutrition supplements to boost immunity changed?	0.0005
Q. 25	During the COVID-19 pandemic, how has the support of your family and friends in eating healthy changed?	0.0362
Q. 29	During the COVID-19 pandemic, how has your sitting and screen time changed?	0.0362
Q. 30	During the COVID-19 pandemic, how have your hours of sleep changed?	0.0010
Q. 23	During the COVID-19 pandemic, how has your intake of immunity-boosting foods (lemon, garlic, turmeric, green leafy vegetables, and citrus fruits) in the diet changed?	0.0278
Q. 28	During the COVID-19 pandemic, how has your participation in leisure and household chores changed?	0.0279
Family Status		
Q. 13	During the COVID-19 pandemic, how has your habit of snacking between meals changed?	0.0019
Q. 22	During the COVID-19 pandemic, how has your consumption of unhealthy food when you are bored, stressed, or upset changed?	0.0201
Socio-economic status		
Q. 12	During the COVID-19 pandemic, how has your probability of skipping one of the main meals (breakfast, lunch, and dinner) changed?	0.0032
Q. 13	During the COVID-19 pandemic, how has your habit of snacking between meals changed?	<0.0001
Q. 14	During the COVID-19 pandemic, how has your quantity and portions of meals and snacks changed?	0.0082
Q. 15	During the COVID-19 pandemic, how has your daily intake of fruits and vegetables changed?	0.0002
Q. 16	During the COVID-19 pandemic, how has your intake of a balanced diet (including healthy ingredients such as whole wheat, pulses, legumes, eggs, nuts, fruits, and vegetables) changed?	<0.0001
Q.18	During the COVID-19 pandemic, how has your consumption of fried food changed?	0.0262
Q. 19	During the COVID-19 pandemic, how has your intake of sugar-sweetened beverages (carbonated soft drinks and sugar-sweetened juices) changed?	0.0385
Q.21	During the COVID-19 pandemic, how has your participation in cooking new and traditional recipes changed?	0.0007
Q. 23	During the COVID-19 pandemic, how has your intake of immunity-boosting foods (lemon, garlic, turmeric, green leafy vegetables, and citrus fruits) in the diet changed?	0.0017
Q. 25	During the COVID-19 pandemic, how has the support of your family and friends in eating healthy changed?	0.0149
Q. 30	During the COVID-19 pandemic, how have your hours of sleep changed?	0.0181
Q. 31	During the COVID-19 pandemic, how have your stress and anxiety levels changed?	0.0440

**Table 7:** Percentages for questions by marital status.

		Significantly Decreased	Slightly Decreased	Grossly Similar	Slightly Increased	Significantly Increased
Q12	Single	13.6%	15.2%	35.4%	19.2%	16.7%
	Married	5.2%	9.7%	42%	24.2%	19%
Q13	Single	7.4%	14.4%	26.8%	27.4%	24%
	Married	4.1%	5.6%	29.4%	34.9%	26%
Q14	Single	7.6%	12.6%	29.3%	28.8%	21.7%
	Married	3%	8.6%	37.6%	31.6%	19.3%
Q16	Single	5.1%	17.7%	40.9%	21.2%	15.2%
	Married	4.8%	9.3%	40.9%	30.5%	14.5%
Q21	Single	5.1%	5.6%	36.4%	28.3%	24.8%
	Married	2.2%	3.7%	40.5%	19.7%	33.8%
Q23	Single	2.5%	3.5%	37.9%	35.4%	20.7%
	Married	0.4%	1.5%	31.2%	36.8%	30.1%
Q24	Single	3%	5.6%	41.4%	31.3%	18.7%
	Married	1.5%	0.7%	36.1%	35.3%	26.4%
Q30	Single	6.6%	12.6%	21.7%	26.3%	32.8%
	Married	6.7%	11.2%	38.7%	24.2%	19.3%

**Table 8:** Percentages for questions by educational status.

Question Number		Significantly Decreased	Slightly Decreased	Grossly Similar	Slightly Increased	Significantly Increased
Q13	High School	14.3%	11.9%	33.4%	21.4%	19%
	Bachelor	3.8%	12%	27%	33.1%	24.2%
	High Education	6.1%	3.8%	32.1%	30.3%	27.8%
Q16	High School	19.1%	11.9%	47.6%	16.7%	4.8%
	Bachelor	4.4%	14.0%	39.9%	26.3%	15.4%
	High Education	1.5%	10.6%	40.9%	30.3%	16.7%
Q24	High School	2.4%	14.3%	28.6%	31.0%	23.8%
	Bachelor	3.1%	1.7%	38.2%	33.5%	23.6%
	High Education	0.0%	1.5%	41.7%	34.9%	22.0%
Q25	High School	9.5%	0.0%	38.1%	23.8%	28.6%
	Bachelor	2.7%	3.4%	45.4%	29.7%	18.8%
	High Education	3.0%	3.0%	41.7%	40.2%	12.1%
Q29	High School	7.1%	2.4%	31.0%	21.4%	38.1%
	Bachelor	5.5%	4.1%	28.0%	25.3%	37.2%
	High Education	0.0%	4.6%	43.2%	22.0%	30.3%
Q39	High School	21.4%	9.5%	28.6%	19.1%	21.4%
	Bachelor	6.1%	11.6%	28.7%	24.6%	29.0%
	High Education	3.0%	12.9%	38.6%	28.0%	17.4%

**Table 9:** Percentages for questions by socio-economic status.

Question Number		Significantly Decreased	Slightly Decreased	Grossly Similar	Slightly Increased	Significantly Increased
Q12	Below average	21.7%	15.2%	21.7%	26.1%	15%
	Average	6%	13.2%	39%	22.3%	19.5%
	Higher than average	11.7%	6.8%	47.6%	19.4%	14.6%
Q13	Below average	21.7%	17.4%	15%	28.3%	17.4%
	Average	3.8%	9.1%	28%	32.4%	26.8%
	Higher than average	2.9%	5.8%	35%	31.1%	25%
Q14	Below average	17.4%	10.9%	39.1%	19.6%	13%
	Average	3.5%	10.4%	32.4%	32.1%	21.7%
	Higher than average	3.9%	9.7%	36.9%	30.1%	19.4%
Q15	Below average	19.6%	13%	34.8%	19.6%	13%
	Average	4.7%	11%	35.5%	34.3%	14.5%
	Higher than average	1.9%	5.8%	50.9%	26.2%	15.1%
Q16	Below average	21.7%	15.2%	39.1%	19.6%	4.4%
	Average	3.8%	15.1%	41.5%	24.8%	14.8%
	Higher than average	1%	4.9%	39.8%	35%	19.4%
Q18	Below average	17.4%	8.7%	34.8%	17.4%	21.7%
	Average	7.6%	16.8%	44.3%	19.3%	12%
	Higher than average	5.9%	9.9%	56.4%	18.8%	8.9%
Q19	Below average	17.4%	13%	28.3%	15.2%	26.1%
	Average	14.5%	13.2%	37.4%	19.2%	15.7%
	Higher than average	12.6%	12.6%	53.4%	13.6%	7.8%
Q21	Below average	15.2%	4.4%	30.4%	28.3%	21.7%
	Average	1.6%	5%	39.6%	24.2%	29.6%
	Higher than average	3.9%	2.9%	39.8%	18.5%	35%
Q23	Below average	8.7%	2.2%	32.6%	34.8%	21.7%
	Average	0.6%	2.8%	33%	37.4%	26.1%
	Higher than average	0.0%	1%	37.9%	33%	28.2%
Q25	Below average	10.9%	8.7%	45.7%	19.6%	15.2%
	Average	3.1%	2.5%	44.7%	32.4%	17.3%
	Higher than average	1%	1.9%	39.8%	36.9%	20.4%
Q30	Below average	15.2%	10.9%	21.7%	23.9%	28.3%
	Average	4.7%	10.7%	31.1%	25.2%	28.3%
	Higher than average	8.7%	15.5%	36.9%	25.2%	13.6%
Q31	Below average	2.2%	0.0%	8.7%	28.3%	60.9%
	Average	0.6%	3.5%	21.4%	34.9%	39.6%
	Higher than average	1.9%	3.9%	28.2%	33%	33%

**Table 10:** Pearson correlation with *p*-values between questions.

Question Number	Question	Correlation Value	*p*-Value
Q14. During the COVID-19 pandemic, how has your quantity and portions of meals and snacks changed?			
Q10	During the COVID-19 pandemic, how has your weight changed?	0.50	<0.0001
Q13	During the COVID-19 pandemic, how has your habit of snacking between meals changed?	0.71	<0.0001
Q18	During the COVID-19 pandemic, how has your consumption of fried food changed?	0.48	<0.0001
Q22	During the COVID-19 pandemic, how has your consumption of unhealthy food when you are bored, stressed, or upset changed?	0.48	<0.0001
Q16. During the COVID-19 pandemic, how has your intake of a balanced diet (including healthy ingredients such as whole wheat, pulses, legumes, eggs, nuts, fruits, and vegetables) changed?			
Q15	During the COVID-19 pandemic, how has your daily intake of fruits and vegetables changed?	0.58	<0.0001
Q18. During the COVID-19 pandemic, how has your consumption of fried food changed?			
Q17	During the COVID-19 pandemic, how has your consumption of junk food and fast food changed?	0.58	<0.0001
Q19	During the COVID-19 pandemic, how has your intake of sugar-sweetened beverages (carbonated soft drinks and sugar-sweetened juices) changed?	0.51	<0.0001
Q22	During the COVID-19 pandemic, how has your consumption of unhealthy food when you are bored, stressed, or upset changed?	0.51	<0.0001
Q20. During the COVID-19 pandemic, how has your consumption of sweets, candies, and chocolate changed?			
Q19	During the COVID-19 pandemic, how has your intake of sugar-sweetened beverages (carbonated soft drinks and sugar-sweetened juices) changed?	0.56	<0.0001
Q22	During the COVID-19 pandemic, how has your consumption of unhealthy food when you are bored, stressed, or upset changed?	0.51	<0.0001
Q25. During the COVID-19 pandemic, how has the support of your family and friends in eating healthy changed?			
Q24	During the COVID-19 pandemic, how has your intake of nutrition supplements to boost immunity changed?	0.49	<0.0001
Q26	During the COVID-19 pandemic, how has your interest in learning healthy eating tips from the media (newspaper articles, magazines, blogs, videos, T.V. shows, and text messages) changed?	0.52	<0.0001

**Table 11:** Significant pairwise comparisons from the Tukey–Kramer HSD test.

Level (Mean ± SD)	Level (Mean ± SD)	Difference 95% CI	Backtransformd Difference 95% CI	*p*-Value
2 (3.56 ± 0.40)	5 (3.36 ± 0.31)	0.202 (0.05,0.36)	1.224 (1.05,1.43)	0.0041
3 (3.53 ± 0.34)	5 (3.36 ± 0.31)	0.172 (0.05,0.29)	1.188 (1.05,1.34)	0.0008
3 (3.53 ± 0.34)	4 (3.41 ± 0.36)	0.122 (0.01,0.24)	1.130 (1.01,1.27)	0.0406

## Data Availability

The data presented in this study are available upon reasonable request from the corresponding author.
